# An auditory multiclass brain-computer interface with natural stimuli: Usability evaluation with healthy participants and a motor impaired end user

**DOI:** 10.3389/fnhum.2014.01039

**Published:** 2015-01-09

**Authors:** Nadine Simon, Ivo Käthner, Carolin A. Ruf, Emanuele Pasqualotto, Andrea Kübler, Sebastian Halder

**Affiliations:** ^1^Institute of Medical Psychology and Behavioral Neurobiology, University of TübingenTübingen, Germany; ^2^Max Planck Institute for Intelligent SystemsTübingen, Germany; ^3^Institute of Psychology, University of WürzburgWürzburg, Germany; ^4^Psychological Sciences Research Institute, Université Catholique de LouvainLouvain-la-Neuve, Belgium

**Keywords:** P300, EEG, auditory BCI, brain-computer interface, communication, ALS

## Abstract

Brain-computer interfaces (BCIs) can serve as muscle independent communication aids. Persons, who are unable to control their eye muscles (e.g., in the completely locked-in state) or have severe visual impairments for other reasons, need BCI systems that do not rely on the visual modality. For this reason, BCIs that employ auditory stimuli were suggested. In this study, a multiclass BCI spelling system was implemented that uses animal voices with directional cues to code rows and columns of a letter matrix. To reveal possible training effects with the system, 11 healthy participants performed spelling tasks on 2 consecutive days. In a second step, the system was tested by a participant with amyotrophic lateral sclerosis (ALS) in two sessions. In the first session, healthy participants spelled with an average accuracy of 76% (3.29 bits/min) that increased to 90% (4.23 bits/min) on the second day. Spelling accuracy by the participant with ALS was 20% in the first and 47% in the second session. The results indicate a strong training effect for both the healthy participants and the participant with ALS. While healthy participants reached high accuracies in the first session and second session, accuracies for the participant with ALS were not sufficient for satisfactory communication in both sessions. More training sessions might be needed to improve spelling accuracies. The study demonstrated the feasibility of the auditory BCI with healthy users and stresses the importance of training with auditory multiclass BCIs, especially for potential end-users of BCI with disease.

## Introduction

One of the main goals in the development of brain computer interfaces (BCIs) is the implementation of devices that can serve as communication aids for severely paralyzed persons Most common BCIs rely on visual stimulation and the patient's ability to control eye movements (Birbaumer and Cohen, 2007). However, neurological disorders, such as stroke or traumatic brain injury, and neurodegenerative diseases, such as amyotrophic lateral sclerosis (ALS), can lead to a complete locked-in syndrome (CLIS). In this condition, control over all muscles (including eye muscles) is lost (Bauer et al., 1979). Since auditory information processing is not affected in persons with ALS (Murguialday et al., 2011) auditory BCI systems could be the key to communication in CLIS. In recent years there have been several approaches that focused on the auditory modality when developing new BCI systems.

Auditory paradigms using the modulation of slow cortical potentials (SCPs) and sensorimotor rhythms (SMRs) as input signal did not yield satisfactory results (Pham et al., 2005; Nijboer et al., 2008a). Only a minority of participants achieved classification accuracies above 70%, which was marked as a threshold for successful BCI communication (Kübler et al., 2001). Further, several studies implemented paradigms based on event-related potentials (ERPs) that allow for binary communication (Hill et al., 2004, 2005; Halder et al., 2010; Hill and Schoelkopf, 2012; Lopez-Gordo et al., 2012). While tests with healthy participants yielded satisfactory results, a spelling solution based on a binary choice paradigm would be slow and these paradigms have not been tested with target end users (Halder et al., 2010).

Therefore, we implemented and evaluated a multiclass BCI based on ERPs as control signals. A number of previous studies used a modification of the P300 speller introduced by Farwell and Donchin (1988), in which the rows and columns of a letter matrix are coded by tones (Sellers and Donchin, 2006; Furdea et al., 2009; Klobassa et al., 2009; Käthner et al., 2013). These studies are presented below along with other approaches that influenced the design of the paradigm presented in this paper.

Furdea et al. (2009) proposed an auditory P300 speller using a 5 × 5 letter matrix. The rows were coded by the spoken numbers 1 to 5, columns by the numbers 6 to 10. To choose a letter, participants first had to attend to the number representing the target row, and subsequently to the number coding the desired column. Out of 13 healthy participants, nine were able to write a word with accuracies of 70% or higher (mean 65%). The comparatively long stimulus duration of the spoken numbers (450 ms) yielded a mean information transfer rate (ITR) of 1.54 bits/min. In the evaluation of this paradigm with four persons with LIS caused by ALS, accuracies higher than chance level have been achieved, but the mean rate of correctly chosen letters was only 12% (Kübler et al., 2009).

Klobassa et al. (2009) used shorter (110 ms) and natural tones (e.g., the chime of a clock) to represent rows and columns of a 6 × 6 matrix. The authors reported mean online classification accuracies of 59%, and offline accuracies of 70%. Mean ITR was 1.86 bits/min. Further, there was a training effect resulting in higher classification accuracies after 11 sessions.

In recent years, it was proposed by Schreuder et al. (2010) to use additional spatial cues to improve auditory ERP based BCIs. In their study, five tones differing in pitch were presented using five speakers placed in front of the participants in a semi-circle. A predefined tone from a certain direction served as target. When averaging across 12 repetitions, binary classification rates of more than 90% and a bit rate of up to 17.39 bits/min was achieved. Mean classification accuracy dropped to 70% when tones were presented without spatial information. In a subsequent study, the paradigm was used for spelling (Schreuder et al., 2011). To choose a letter, participants had to focus attention on one of six different tones that were presented via six speakers placed in a circle around the participants. Each tone/direction represented one group of letters. After choosing one group by focusing attention to the corresponding tone, the single letters of this group were assigned to one tone/direction each. With this method, a maximum of 0.94 letters per minute could be selected. Mean classification accuracies of 77% and bit rates of 5.26 bits/min were achieved.

Directional cues were also used in the study by Höhne et al. (2011) that were presented via headphones. The columns of a 3 × 3 matrix were coded by directional cues (left, both, right speaker) and the rows by pitch (high middle, low), thereby creating 9 distinct tones. While on average 0.89 letters/minute could be chosen, with a mean ITR of 3.4 bits/min, participants described the artificial tones as unpleasant and they were often confused. Subsequently, natural stimuli (sung syllables) were implemented to improve classification performance (Höhne et al., 2012).

Käthner et al. (2013) also proposed a practical multiclass BCI with directional cues. A 5 × 5 letter matrix served as visual aid (visual support matrix). Rows and columns were represented by five artificial tones to allow for fast stimulus presentation. Using interaural time difference (ITD) and interaural level difference (ILD), directional cues were added to the tones to improve discriminability. Tones were presented via headphones to simplify the set-up. To select a letter, participants first had to attend to the tone that coded the column, and after a short break to the tone that coded the row. Different interstimulus intervals were tested. In the best case, an average classification accuracy of 66% with a bitrate of 2.05 bits/min was achieved.

### Study aims

A major goal of the present study was to further improve the auditory P300 speller with directional cues proposed by Käthner et al. (2013). Since it was shown that the use of natural stimuli is advantageous (Höhne et al., 2012), natural tones were implemented to improve classification accuracies. The present paradigm was first tested with healthy participants and subsequently validated with a person diagnosed with ALS.

Since Klobassa et al. (2009) reported an increase in accuracy over several sessions using an auditory BCI, two sessions were performed to reveal a possible training effect that would be evident in better performance and possibly in a higher amplitude/shorter latency of the P300. Further, we investigated if the use of natural sounds can reduce the subjective workload compared to the previous study and if a positive influence of motivation and mood on performance and P300 characteristics could be found.

## Methods

### Participants

Originally, 14 healthy participants were recruited for the study. One had to be excluded due to insufficient hearing and two due to technical problems during the measurements. Thus, 11 healthy subjects (8 female, mean age 24.27 years, *SD* = 7.14 years, 2 left handed) and one participant with ALS (female, age 66) were included in the study. Healthy subjects had no experience with auditory BCIs and no or little experience with other kinds of BCIs. They were compensated with course credits. All of them were students (nine of them students of psychology) with German as their mother tongue. Only healthy subjects with no history of neurological or psychological disorders, difficulties localizing sounds in space, and no general hearing problems were included in the study. To rule out hearing impairments, a test based on the Hughson-Westlake method (conforms to ISO 8253; Madsen Xeta Audiometer, GN Otometrics, Denmark) was conducted. All participants gave informed consent prior to the experiment. The study was approved by the Ethical Review Board of the Medical faculty of the University of Tübingen.

The participant with ALS had no experience with auditory BCIs. She was right handed and reported not to be musical. The disease was diagnosed at the age of 63 as being sporadic with bulbar onset. Her husband took care of her at home, where the experiments were conducted. At the time of the measurements, the patient was still able to talk, but difficult to understand. Her ability to move around was heavily restricted. To walk within the flat she relied on a walking frame, outside on a wheelchair. At the time of the first measurement the patient reached a score of 17.5 (ranging from 48, no impairment, to 0, locked in) on the ALS Functional Rating Scale-Revised (ALSFRS-R, Cedarbaum et al., 1999). This score had decreased to 16 at the time of the second measurement, 1 month later. Since the hearing threshold of the patient was very high, the volume of the stimuli was adjusted and it was ensured that she was able to perceive and discriminate correctly all the stimuli.

### Data acquisition and material

Participants were seated in a comfortable chair, about 0.5–1 m from a 17″monitor. Data acquisition, processing, and storage were conducted on an IBM Thinkpad (Intel Core Duo 2.53 GHz, 1.89 GB RAM, Microsoft Windows XP SP3 Professional). Data acquisition and stimuli presentation was controlled by the BCI2000 software (Schalk et al., 2004) in combination with the Brain Vision Recorder (Version 1.2, Brain Products GmbH, Deutschland). Auditory stimuli were presented via circumaural headphones (Sennheiser 280 HD Pro) to minimize background noise.

The EEG was recorded using 28 active Ag/AgCl electrodes (Easycap GmbH, Germany) following the modified version of the international 10–20 system of the American Electroencephalographic Society (Sharbrough et al., 1991). Electrodes were placed at positions F3, Fz, F4, C5, C3, C1, Cz, C2, C4, C6, CP5, CP3, CP1, CPz, CP2, CP4, CP6, P3, P1, Pz, P2, P4, PO7, PO3, POz, PO4, PO8, and Oz. Channels were referenced to the left mastoid and grounded to the right. Electrooculogram was recorded with four electrodes placed below and above the right eye and at the outer canthi of both eyes. Signals were amplified with a sampling rate of 500 Hz using a BrainAmp DC amplifier (Brain Products GmbH, Germany). Signals were filtered with a high pass of 0.1 Hz, a low pass of 30 Hz and a notch filter at 50 Hz. These were the same electrode positions and settings that were used in Käthner et al. (2013) to allow for a better comparison across studies.

### Letter matrix

**Figure 1:**
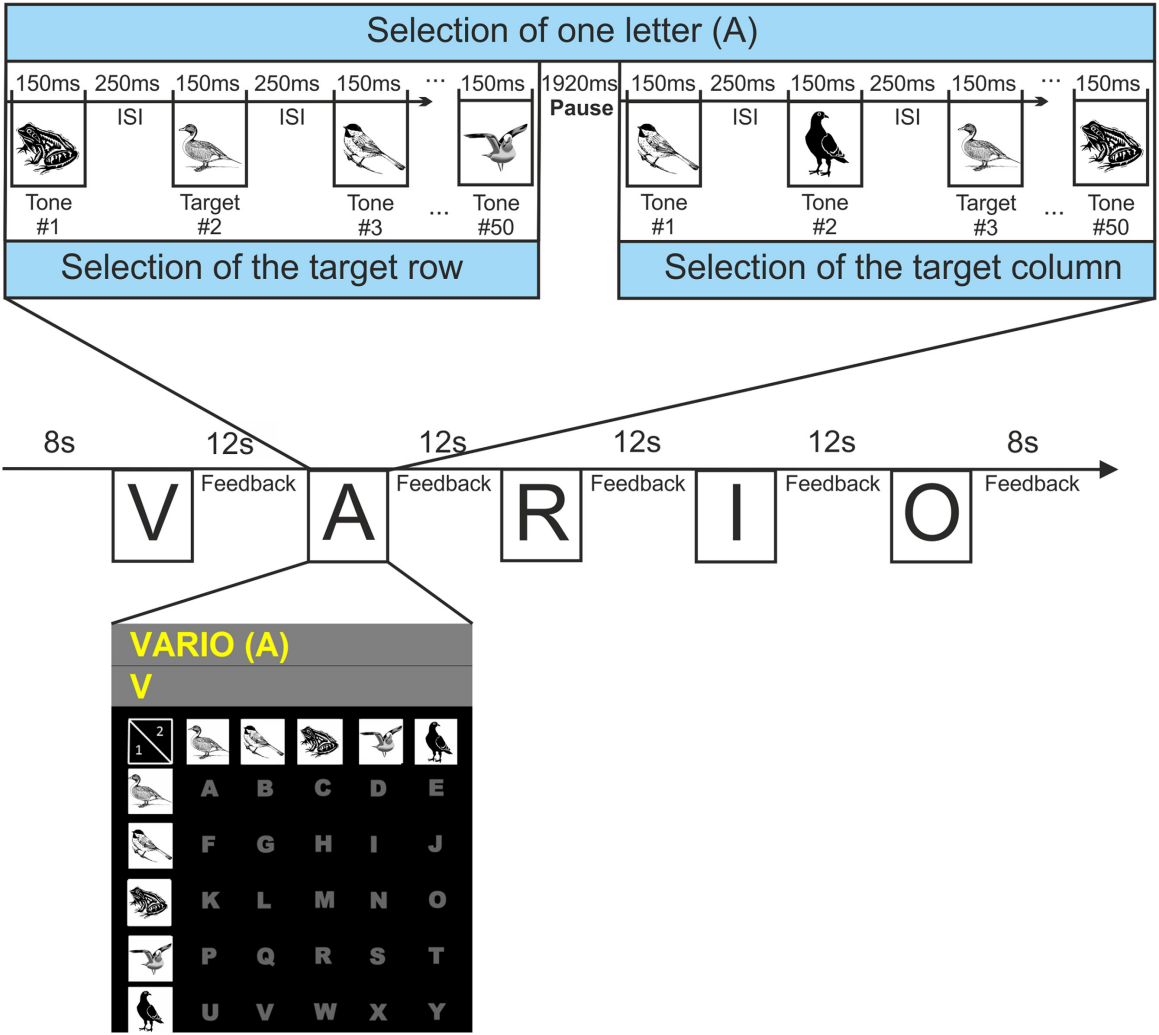
**Visualization of the tasks needed to spell a specific word**. To select a particular letter (in this example the letter A), participants had to first focus attention on the tone coding the target row and in the second step, concentrate on the tone coding the target column (twice duck in the example). The tones were played in random order and the sequence of all tones was repeated a total of 10 times. The matrix was displayed to the participants during spelling. For copyright reasons the displayed animal illustrations differ from those used in the experiment.

During the measurement, the participants were facing a screen displaying a 5 × 5 letter matrix with the letters A to Y (see Figure 1). Since the study used an auditory paradigm the matrix only served as static visual aid. Each row and each column of the matrix was coded by one of five animal tones (shown in the visual support matrix). The numbers “1” and “2” in the top left corner of the matrix indicated the order of selection of rows and columns (during the experiment first the tones coding the rows were presented, then the tones coding the columns). The words that had to be written were presented in a row above the matrix, with the current letter presented in brackets. In a second row the letters selected during online spelling were fed back to the participants.

### Auditory stimuli

**Figure 2:**
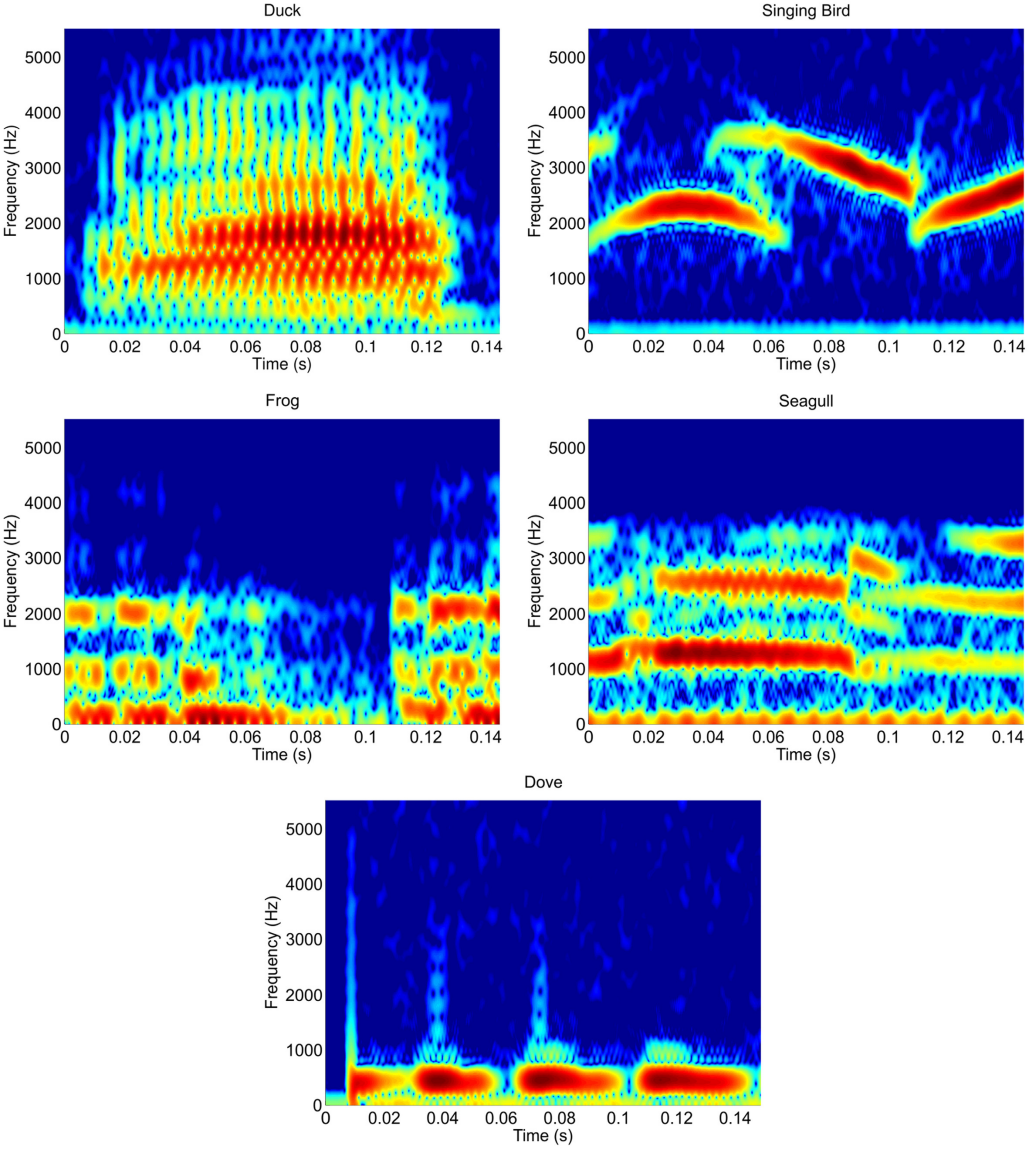
**Spectrograms of the auditory stimuli**.

**Figure 3:**
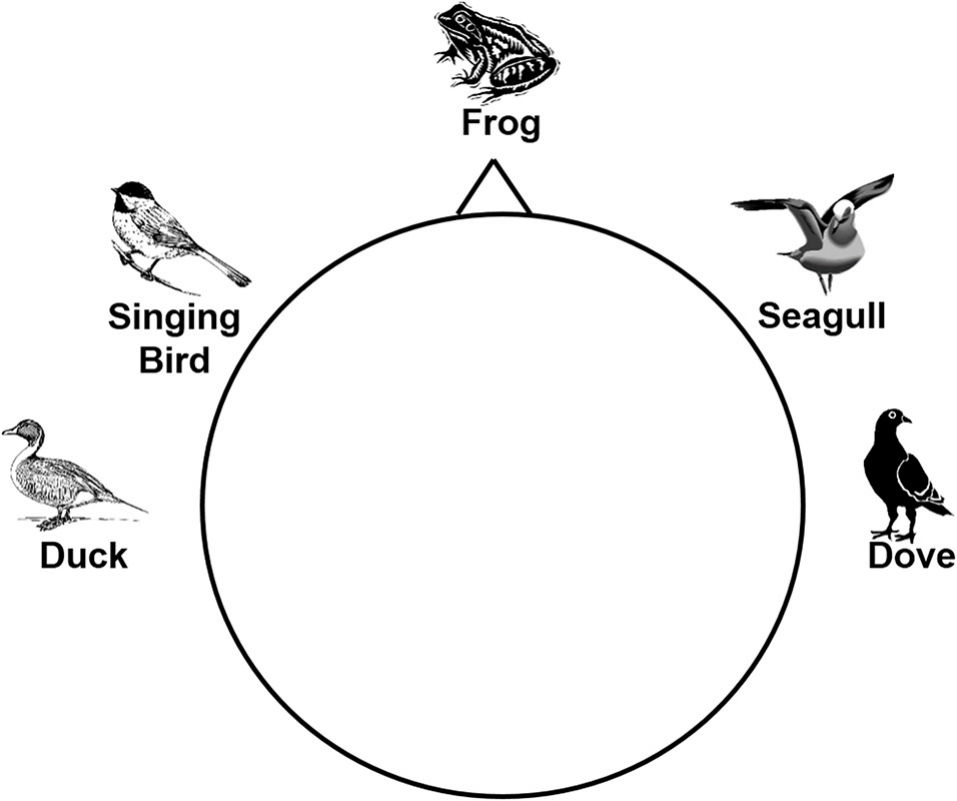
**Visualization of the simulated sound sources for the five animal voices that was presented to the participants during the experiment**.

Rows and columns of the letter matrix were coded by five different tones. In a pre-study five groups of different tones were evaluated to determine the tones that were best discriminable. The groups included either five different white noise tones, five sine tones, five tones of everyday life (e.g., ticking of a clock) or one of two sets of animal voices. Ten subjects rated the discriminability of the tones for each of the five groups on a visual analog scale (VAS)—a 10 cm long horizontal line ranging from 0 to 10. The second group of animal tones (with the sounds of a duck, singing bird, frog, seagull, and a dove) achieved the best ratings of discriminability and was therefore chosen for the experiment. The sounds had been downloaded from the webpage http://www.soft-ware.net/animal-sounds and edited. Animal tones were cut to 150 ms length, using the interval in which the sound was best distinguishable. Figure 2 shows the spectrograms of the sound files, illustrating the heterogeneous temporal structure. To better differentiate the tones via headphones, directional cues were added as described in Käthner et al. (2013). The simulated sound sources were left (duck), center-right (singing bird), center (frog), center-left (seagull), and right (dove). To remind participants of this scheme, the illustration shown in Figure 3 was displayed on a sheet of paper that served as a reference base.

### Procedure

The study consisted of two measurements on consecutive days. To control for circadian effects (Wesensten et al., 1990) both measurements were conducted at the same time of day.

The measurements with the participant with ALS were conducted at her home. Due to a temporary worsening of the participant's state of health, the interval between the two measurements was 1 month.

Before the beginning of the measurements, the sounds were played to the participants a number of times to familiarize them with the task of identifying certain tones. Also the volume could be adjusted. Each tone had a length of 150 ms, followed by an inter stimulus interval (ISI) of 250 ms, thus the stimulus onset asynchrony (SOA) was 400 ms. The choice of the timing parameters was motivated by the results of previous studies which showed that the choice of stimulation speed is crucial for performance with auditory BCIs (Höhne and Tangermann, 2012; Käthner et al., 2013). Höhne and Tangermann (2012) tested SOAs between 50 and 1000 ms in a simple auditory oddball paradigm and found increasing P300 amplitudes with slower stimulation speed. With regard to ITR, SOAs between 87 and 200 ms were optimal for most healthy participants of the study. In their online study using a multiclass BCI paradigm Käthner et al. (2013) could confirm the finding that optimal stimulation parameters differed between participants. On average, the highest ITR was achieved in the condition with an ISI of 400 ms. In both studies, however, stimulus duration of the artificial tones (40 ms) was considerably shorter than the natural stimuli of the present study (150 ms). The stimulus duration probably also influences auditory BCI performance, but has not yet been systematically evaluated. As a tradeoff between speed and accuracy we decided for a SOA of 400 ms, although higher P300 amplitudes and accuracies might be achieved with slower stimulation.

For the selection of one letter, two steps were needed. In the first step, the tone representing the row containing the target letter had to be selected. While all five animal tones were presented 10 times each in pseudo randomized order, the participant had to focus attention on the target tone and count its appearance. After a pause of 1.92 s, the tones representing the columns were presented. Then the participants had to concentrate on the tone representing the column with the target letter. Between letter selections there was a pause of 12 s to focus on the next letter. The process of letter/word selection is illustrated in Figure 1. A recording of one exemplary trial is published as Supplementary Material.

#### Session 1

For the training of the classifier, participants completed three calibration runs in which they had to select the letters “AGMSY.” Based on these runs, feature weights were calculated for the online classification. After training the classifier, nine words had to be written in an online copy spelling task during which feedback about the selected letters was provided (see Table 1). No feedback about the selected row (necessary sub-step for one letter selection) was provided. Each row and each column was represented once within each word to minimize possible confounds caused by specific tones or directions. The copy spelling was divided into three blocks with three words each and breaks between the blocks.

**Table 1:** **Blocks of letters that had to be spelled during the two sessions**.

	**Session 1 online**	**Session 2 online**	**Session 2 offline**
Calibration	AGMSY	Classification weights of Session 1	VARIO
	AGMSY		GRUEN
	AGMSY		HUNGER
Letters to be spelled	15	15	16
Copy spelling	VARIO	VARIO	
	GRUEN	GRUEN	
	HUNGER	HUNGER	
	TUMBI	TUMBI	TUMBI
	RUBIO	RUBIO	RUBIO
	VALERI	VALERI	VALERI
	UMBIT	UMBIT	UMBIT
	PHLEX	PHLEX	PHLEX
	VIRAGO	VIRAGO	VIRAGO
Letters to be spelled	48	48	32
Free spelling	5 letter word of own choice	BRAIN POWER	BRAIN POWER
Letters to be spelled	5	10	10

After the copy spelling tasks and a short break, participants completed a free spelling task. They were asked to think of a five letter word that they wanted to write. Before the actual spelling, they wrote the desired word on a piece of paper and revealed it only after they had completed the spelling.

The course of the measurement was the same for the participant with ALS, with the exception that she was allowed to take breaks between spelling of individual words. Since she was physically too exhausted to perform the free spelling, the session was stopped after the copy spelling.

#### Session 2

At the beginning of Session 2, the task was explained to the participants and they could listen to the tones again. For the healthy participants, there was no distinct calibration run in Session 2. Since no changes in the individual parameters of the participants within 2 days were expected (Nijboer et al., 2008b), classification weights of Session 1 were applied. The procedure of the online copy spelling was the same as in the first session. In the subsequent free spelling task the participants had to spell the words BRAIN and POWER (no display of the words to spell).

Since the analysis of the data of the healthy participants revealed that the missing calibration run in Session 2 lead to a decline in accuracy, a distinct calibration run was conducted with the participant with ALS. As in Session 1, she completed three calibration runs followed by the copy spelling tasks.

### Questionnaires

At the beginning of the first session all participants completed a demographic questionnaire which also assessed the musicality of the participants as well as their experience with BCIs (and auditory BCIs in particular). Handedness was assessed using the Edinburgh Handedness Inventory (Oldfield, 1971). Prior to each measurement, participants rated their mood and motivation on VAS. The 10 cm long lines of the VAS ranged from “0 = not motivated at all” to “10 = extremely motivated” and from “0 = extremely bad mood” to “10 = extremely good mood,” respectively. For the assessment of general functioning the participant with ALS completed the ALS function rating scale (Cedarbaum et al., 1999) prior to each measurement session.

Subsequent to each training session participants completed an electronic version of the NASA Task Load Index (NASA-TLX, NASA Human Performance Research Group, 1987). The NASA-TLX measures the subjective workload during human machine interaction. On the basis of six subscales (Mental, Physical and Temporal Demands, Own Performance, Effort, and Frustration) a global value for the subjective workload is determined. Each of the six factors are first evaluated on a 21-point scale ranging from “very low” (0) to “very high” (100; for the factor Own Performance the scale ranged from “bad” to “good”). In a second step, pairwise comparisons of all the factors are used to determine to what degree each of the factors contributed to the overall workload of the task. This procedure yields a total value between 0 and 100, with 100 representing the highest subjective work load. The NASA-TLX is a validated, sensitive and reliable instrument for measuring the subjective workload (Rubio et al., 2004; Hart, 2006). Following the user-centered design (ISO 9241-210) it has been introduced as a measure of usability, specifically efficiency which relates accuracy to the costs involved in using the technology (Zickler et al., 2011; Kübler et al., 2014).

At the end of the second session, participants completed the System Usability Scale (SUS, Brooke, 1996). On the basis of 10 questions (concerning topics like complexity of the application, need of support and training) the subjective usability of a system is assessed. Questions are answered on a five point Likert scale ranging from “1 = Strongly disagree” to “10 = strongly agree.” The completion of the SUS results in a total value between 0 and 100, with 100 representing the highest subjective usability. The SUS is a reliable instrument that correlates high with other measures of usability (Brooke, 1996).

Further, participants were asked to evaluate their experiences with the auditory P300 speller with a custom-made questionnaire. They responded to 13 statements on a five point Likert scale ranging from “1 = Agree” to “5 = Disagree.” The questionnaire also offered space for suggestions on how to improve the system.

The participant with ALS was unable to write due to her disease, therefore, she answered the questions orally with the help of her husband.

### Online signal classification

Classification weights were calculated in Matlab using stepwise linear discriminant analysis (SWLDA) implemented in the P300 GUI of the BCI2000 software. After the classification parameters had been determined, the EEG data were classified online using BCI2000. The usage of SWLDA is an established procedure in BCI studies and it was shown that this approach surpasses other classification techniques (Krusienski et al., 2006). It determines features from voltage values from each of the 28 electrodes that best discriminate the two classes (targets/non-targets). Firstly, the data was segmented into post stimulus epochs of 800 ms and then moving average filtered and decimated, corresponding to a sampling rate of 20 Hz. This procedure yielded 16 values per stimulus. From this data set, features were selected in a stepwise process. The feature that best predicted the target label using least square regression was added to the discriminant function (*p*-value ≤ 0.1). After adding another feature to the function, a backward stepwise discriminant analysis was performed to remove the features that were no longer significant (*p* > 0.15). This process was repeated until a maximum of 60 features was reached or no additional features met the inclusion/exclusion criteria (Krusienski et al., 2006).

### Offline data analysis

The EEG data was analyzed with Matlab (version R2012b) in combination with the EEGLab toolbox (version 10.2.2.4b; Delorme and Makeig, 2004). It was segmented using an epoch of 800 ms, starting at the onset of a stimulus and baseline corrected using a pre-stimulus interval of 200 ms. Averages and grand averages over all runs were calculated for each participant for targets and non-targets. Data was only analyzed for the copy spelling runs. Because the missing calibration run in Session 2 lead to a decline in online performance (see BCI Performance), the classifier was retrained offline using the first three words acquired during the copy-spelling runs of that session (VARIO, GRUEN, and HUNGER) and feature weights were applied for classification of the remaining words. Averages and grand averages were calculated for all nine words.

Statistical analysis of ERPs was restricted to Pz, where the P300 is prominent. The maximum positive peak between 250 and 560 ms was defined as P300. Topographies of differential ERP activity (targets minus non-targets) were calculated. For the participant with ALS, data was re-referenced offline using common average referencing, because of artifacts in the data, which were probably caused by technical issues with the reference electrode.

Classification accuracies were calculated as the number of correctly selected letters. Statistical analysis of the data was conducted with SPSS. All statistical analyses were only performed for accuracies achieved in the copy spelling runs. A Wilcoxon signed-rank test was conducted to reveal differences in the accuracies between the 2 days and to check for differences in the online and offline accuracies of the second day.

The error distribution for individual tones was analyzed and depicted in confusion matrices. Chi-square tests were calculated to check the distributions of errors and false positive selections for individual tones. Kendall's Tau was calculated to reveal statistical correlations between questionnaire values (mood, motivation), BCI performance and P300.

### Information transfer rate

ITR was calculated with the formula originally proposed by Shannon and Weaver (1964) and suggested for BCI research by Wolpaw et al. (2000) for online and offline classification accuracies. The bits per minute (bits/min) were computed taking into account the 12 s pause between the selections of individual letters.

## Results

### BCI performance

Classification accuracies and bitrates of all participants are listed in Table 2. Mean online classification accuracies (69.64 ± 13.64%) of Session 2 were significantly smaller than those calculated offline (90.18 ± 9.29%) using the first three copy spelling words from Session 2 to retrain the classifier (*Z* = −2.67, *p* < 0.01). Based on this finding we decided to use the offline results of Session 2 to investigate a possible training effect (see Limitations for a discussion). All reported analyses regarding Session 2 are related to offline results.

**Table 2:** **Classification accuracies and bitrates of all healthy participants**.

**VP**	**Copy spelling**				**Free spelling**			
	**Mean accuracy**		**Bitrate**		**Mean accuracy**		**Bitrate**	
	**Session 1 (in %)**	**Session 2 (in %)**	**Session 1 (bits/min)**	**Session 2 (bits/min)**	**Session 1 (in %)**	**Session 2 (in %)**	**Session 1 (bits/min)**	**Session 2 (bits/min)**
1	82	94	3.49	4.50	80	90	3.34	4.14
2	91	91	4.22	4.22	100	100	5.17	5.17
3	58	88	1.93	3.97	40	90	1.03	4.14
4	83	75	3.57	2.99	80	60	3.34	2.05
5	78	75	3.20	2.99	100	100	5.17	5.17
6	21	100	0.31	5.17	40	100	1.03	5.17
7	94	100	4.50	5.17	100	100	5.17	5.17
8	72	88	2.79	3.97	60	90	2.05	4.14
9	98	97	4.91	4.80	100	100	5.17	5.17
10	77	84	3.13	3.65	40	90	1.03	4.14
11	90	100	4.14	5.17	80	100	3.34	5.17
*M*	76.73	90.18	3.29	4.23	74.55	92.73	3.26	4.51
*SD*	21.63	9.27	1.30	0.81	25.44	11.91	1.76	0.96

The accuracies were calculated as the percentage of correct letter selections. Accuracies of Session 2 were calculated offline.

Classification accuracies in Session 2 (90.18%) were significantly higher than in Session 1 (76.73%; *Z* = −1.99, *p* < 0.05). Average bitrates were 3.29 bits/min in Session 1 and 4.23 bits/min in Session 2.

The participant with ALS reached an accuracy of 20% (± 9.67%) in the first session that rose to 47% (±20.05%) in the second session. This equals bitrates of 0.28 bits/min in session 1 and 1.35 bits/min in Session 2. The classification accuracy of the single words ranged from 0 to 33% in Session 1 and from 20 to 80% in Session 2.

### Multiclass accuracy

To reveal differences in classification accuracies of individual tones, confusion matrices for all 5 tones are shown in Table 3 for healthy participants. In Session 1, 87% of multiclass decisions (row or column selected) were correct compared to 95% in Session 2. In Session 1, the number of total errors for individual tones ranged from 14 errors (dove) to 37 errors (duck). The distribution is a significant deviation from the number of errors that would be expected by chance (28.2 errors per tone; χ^2^ = 13.29, *p* = 0.01). For the multiclass error distribution of individual tones, chi-square tests did not yield significant results, however, a trend toward significance (χ^2^ = 7.56, *p* = 0.056) was revealed for the singing bird sound, where multiclass errors were lowest for duck, four errors, and highest for seagull, 14 errors. It can be inferred from Table 3 that the lowest false positive rate was obtained for the duck sound (19 false positive selections) and the highest for the dove sound (45 false positive selections). A chi-square test revealed a significant deviation from the distribution of false positive selections that would be expected by chance (28.2 false positives per tone, χ^2^ = 15.14, *p* = 0.004). For the distribution of false positive selections for individual tones, a significant result was only obtained for the seagull sound (χ^2^ = 8.93, *p* = 0.03), which was most often wrongly selected if the singing bird was the target sound. The low number of errors in Session 2 does not allow to calculate chi-square values for error and false positive distributions.

**Table 3:** **Confusion matrices for Session 1 and 2 for healthy participants**.

**Target**	**Selected**					**Accuracy (in %)**
	**Duck**	**Bird**	**Frog**	**Seagull**	**Dove**	
**SESSION 1**						
Duck	**172**	8	6	8	15	82
Bird	4	**206**	6	14	12	85
Frog	6	10	**178**	4	11	85
Seagull	7	5	4	**175**	7	88
Dove	2	3	5	4	**184**	93
False positive (in %)	9.95	11.21	10.55	14.63	19.65	**87**
**SESSION 2 (OFFLINE)**						
Duck	**136**	1	2	2	2	95
Bird	3	**156**	0	3	3	95
Frog	4	1	**118**	5	4	89
Seagull	1	2	1	**127**	1	96
Dove	0	1	1	0	**130**	98
False positive (in %)	5.56	3.11	3.28	7.30	7.14	**95**

Classification accuracies of the single tones are shown in the right column, false positives in the bottom row, correct classifications (hits) are in printed in bold.

### ERP analysis

**Figure 4:**
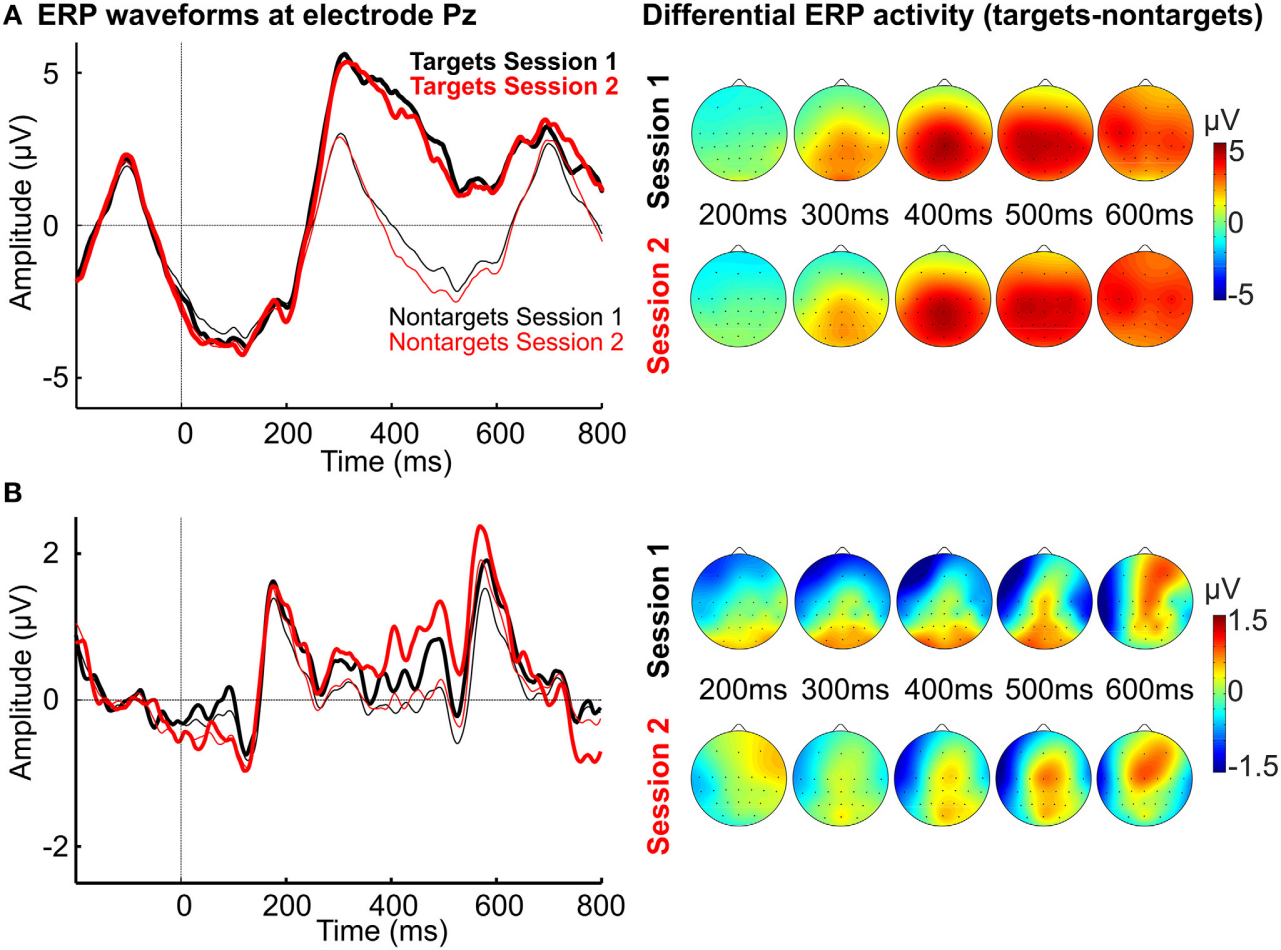
**(A)** Average waveforms for targets (thick lines) and non-targets (thin lines) at Pz for healthy participants and scalp plots of differential ERP activity (targets minus non-targets) for different time points. **(B)** Average waveforms for targets (thick lines) and non-targets (thin lines) at Pz for the participant with ALS and scalp plots of differential ERP activity (targets minus non-targets) for different time points.

Figure 4A shows the average waveforms for targets and non-targets in Session 1 and 2 for the copy spelling task for the healthy participants. Neither peak amplitude of the P300 differed between Session 1 (6.66 ± 3.22 μV) and Session 2 (7.17 ± 2.75 μV; *Z* = 0, *p* = 1) nor was there a significant difference between P300 latencies of Session 1 and 2 (366.59 ± 78.59 ms, 359.59 ± 81.45 ms; *Z* = −1.07, *p* = 0.286). Figure 4A also depicts the grand average spatial distribution of the differential ERP activity (targets minus non-targets) for different time points.

Figure 4B shows the average waveforms and the spatial distribution of the differential ERP activity for the participant with ALS. Peak amplitude at Pz for Session 1 was 0.84 μV at 491.32 ms and 1.35 μV for Session 2 at 493.21 ms.

### Influences of motivation, mood, and musicality

Healthy participants had a mean motivation score of 7.75 (*SD* = 1.73) in Session 1 and 7.70 (*SD* = 1.55) in session. The mean mood score was 6.59 (*SD* = 1.21) in Session 1 and 6.32 (*SD* = 1.24) in Session 2. In both sessions, there was no significant correlation of motivation and mood with classification accuracies, P300 amplitude and latency.

The participant with ALS indicated a motivation of 3.95 in Session 1, and 6.7 in Session 2. The indicated mood score was 6.45 in Session 1, and 8.75 in Session 2.

### Subjective workload

The average score of the NASA-TLX for the healthy participants did not differ significantly between Session 1 (62.24 ± 10.54, range: 43.33–85.00) and Session 2 (69.42 ± 10.59, range: 46.33–83.00; −1.824, *p* = 0.068).

The participant with ALS had a general workload score of 69 in Session 1 and 23.33 after Session 2. The global workload in Session 1 was to a large degree due to the high physical strain as revealed by the subscale physical effort (after the weighting procedure the subscale contributed 32 of the 69 points).

### System usability scale

**Figure 5:**
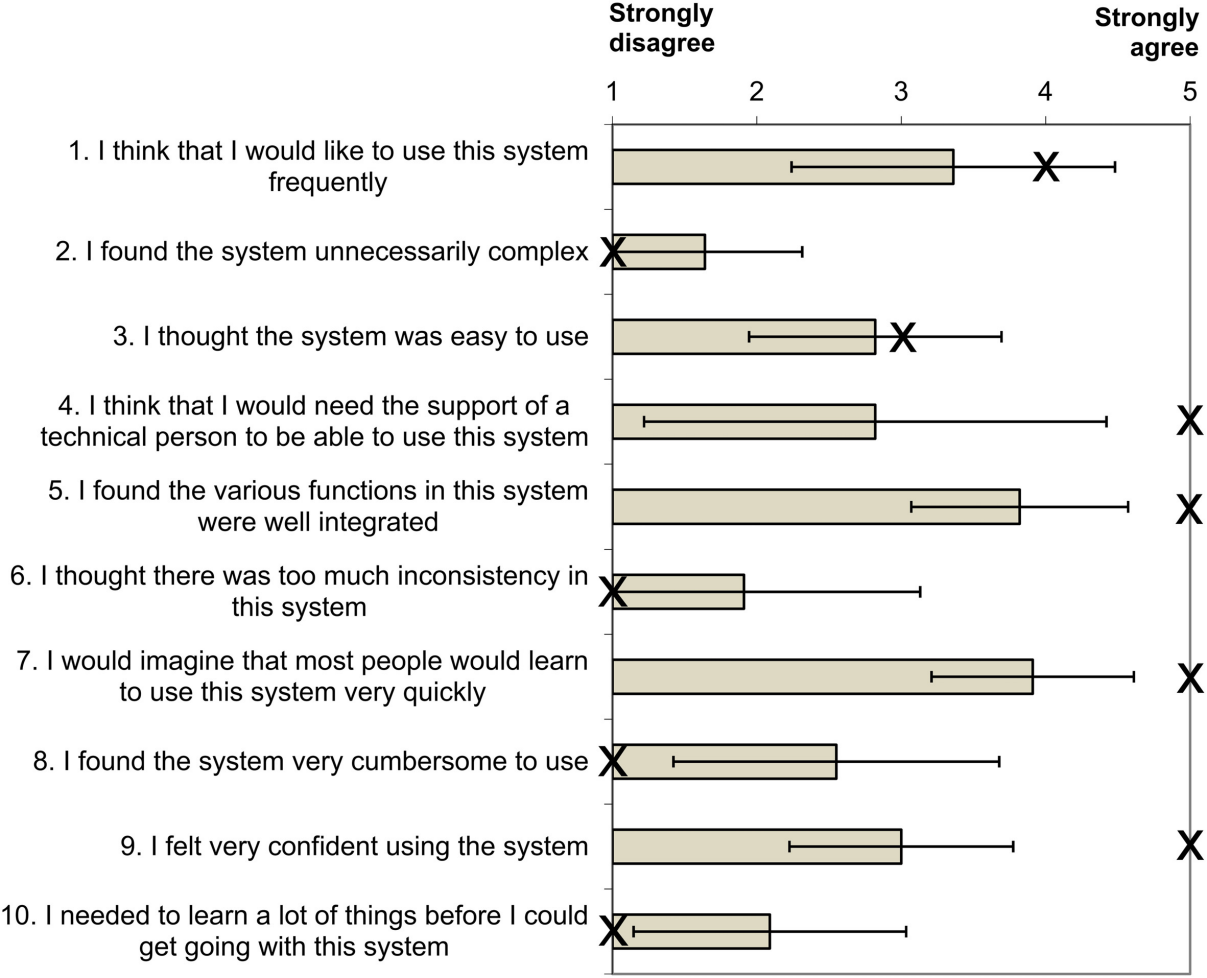
**Scores of the study participants for the items of the system usability scale (mean ± standard deviation)**. The answers of the participant with ALS are marked with an X.

The usability of the system was measured on the basis of the SUS. The global scores can range between 0 and 100, with 100 reflecting the highest usability. At the end of Session 2, answers of the healthy participants yielded individual SUS scores between 47.5 and 80, with a mean SUS score of 64.8 (*SD* = 11.8) across all healthy participants. Figure 5 depicts the responses of the participants to the individual items of the scale.

The statements of the participant with ALS yielded a total SUS score of 82.5. She reported that for item 3 “I thought the system was easy to use” she only marked “3” (moderate agreement) because of the high technical effort that is necessary for the measurement. Apart from that she did not find it difficult to use the BCI speller.

### Post-study evaluation

In the post-study evaluation, 3 out of the 11 healthy participants stated the wish for longer pauses between the presentations of individual tones. Two participants stated that they had problems differentiating the duck and the dove tones. Five reported difficulties in the differentiation of the gull and the singing bird tone. One participant suggested to minimize the number of tone repetitions per row and column.

The participant with ALS suggested to simplify the technical effort that is currently necessary to use the speller. She also suggested to reduce the number of birds in the animal tones and to replace them by other animal voices.

## Discussion

With the proposed auditory multiclass BCI using natural stimuli and directional cues, high mean accuracies could be achieved by healthy participants. Accuracies increased from 76% in the first to 90% in the second session.

### BCI performance

In the second session, healthy participants achieved average spelling accuracies of 90% and all participants achieved accuracies of more than 75% correctly selected letters. Thus, the BCI performance is higher than in the study by Klobassa et al. (2009), in which average accuracies of 70% were calculated offline for the last of 11 training sessions. In the study by Höhne et al. (2011) similarly high accuracies (90%) were achieved within one session, but with a paradigm that only allows to select from 9 different classes as opposed to 25 in the current study. In terms of selection speed, auditory BCIs still lag behind visual spellers, which report bitrates of up to 50.61 bits/min (Lenhardt et al., 2008) and 106 bits/min (Kaufmann and Kübler, 2014). The auditory speller of the current study allowed for only 1.11 selections per minute, but the bitrates of 3.29 bits/min for Session 1 and 4.23 bits/min for Session 2 are still among the highest reported for auditory BCIs (see Riccio et al., 2012 for a review). Bitrate could be optimized by reducing the pause between letter selections and reducing the number of sequences (fixed to 10 independent of individual performance in this study). Dynamic stopping methods allow for determining the optimal number of sequences and have been successfully applied in ERP spellers to improve spelling speed (e.g., Schreuder et al., 2011; Mainsah et al., 2014; for a comparison see Schreuder et al., 2013).

The average classification accuracies achieved by the participant with ALS (20 and 47%) were higher than chance (4%), but did not reach a sufficient level for communication (≥70%; Kübler et al., 2001). For the first session, the achieved accuracy is in the same range as reported by previous studies that tested an auditory multiclass BCI with motor impaired end users. Kübler et al. (2009) reported accuracies between 0 and 25% for the four study participants. Schreuder et al. (2013) tested the AMUSE paradigm (Schreuder et al., 2010) with a severely impaired end user and reported accuracies below chance level for the first and slightly above chance for the second session. However, spelling accuracy steadily improved up to the fourth session (39%). Thus, more training might also improve spelling accuracy with the present auditory multiclass BCI, in particular if testing the system with end users. It is promising that the participant of our study already achieved accuracies substantially above chance in the first two sessions. Only two sessions were conducted in the present study, but the participant with ALS communicated that she would have participated in further sessions, if her physical condition would have allowed it and rated the overall usability as high.

There are a couple of not directly BCI related factors that could have negatively influenced the results. Among the medication that the participant took during the time of the study was Zopiclone, a hypnotic drug that has similar effects on the central nervous system as benzodiazepines, which were shown to directly influence vigilance and P300 amplitude (Engelhardt et al., 1992; Hayashi, 2000). Possibly better results could have been achieved in the second session if the time between measurements had only been a week as originally planned. Nevertheless, further modifications of the paradigm, the stimulus material or the classifier might be needed for end users to achieve higher accuracies even within the first two sessions.

### Training effects and auditory stimuli

The significant increase in classification accuracies from the first to the second session indicates a training effect. The training effect is not reflected in P300 characteristics for the healthy participants. Since neither P300 amplitude nor latency differed significantly between Session 1 and 2, we assume that the training effect is not due to effects that would modulate the P300, such as increased attention or motivation. Possible explanations for this improvement from Session 1 to 2 are either task specific, i.e., adaptation to the task and BCI in general, or stimuli specific, i.e., an improved ability to discriminate the target tones. In the second session compared to the first session, especially the adaptation to the task might have a large influence. The second explanation (improved ability to discriminate the target tones), might be particularly valid for participants, who have difficulties discriminating the target tones initially. For the participant with ALS and increase in the amplitude of the P300 could be observed (from 0.84 to 1.35 μV). No statistically relevant increase in P300 amplitude could be found for the healthy participants, but a slight increase in the amplitude was apparent from Session 1 (6.66 μV) to 2 (7.17 μV). In any case, the results suggest that training should be conducted with auditory multiclass BCIs, especially when testing with end users is performed.

An important goal of this study was to further improve the speller described in Käthner et al. (2013) by replacing the artificial tones with natural stimuli, since several study participants had criticized the stimuli. Average classification accuracies improved from 66% (reported by Käthner et al., 2013) to 77% (first session of this study). This substantial improvement is also apparent in the increase of the bitrate from 2.76 to 3.29 bits/min (first session of this study). Although stimulus duration was shorter, P300 latency at Pz was higher in the study by Käthner et al. (2013; 487 ms) as compared to results of the current study (367 ms). This supports the assumption that the natural stimuli can be more easily discriminated than the artificial tones. The increase of performance for natural stimuli compared to artificial tones was also demonstrated by Höhne et al. (2012). Hence, we think that the increase of performance is mainly caused by the changes in the stimulus material and not by other factors that could also influence the results (see Limitations). While we tried to find a tradeoff between speed and accuracy for the stimulus timing parameters, they could be further optimized. Höhne and Tangermann (2012) demonstrated that individualized timing parameters could boost performance. Further, the effect of stimulus duration has not yet been systematically evaluated.

The multiclass accuracies are high in both sessions (87 and 95% correct), but false positive rates and errors are not equally distributed for the five tones in Session 1. Thus, some tones (with the highest false positive selection rate, i.e., dove, seagull) might be more distinct than others. An alternative explanation could be a general preference of participants to tones that are presented from the right (in our study dove, seagull). Several studies showed a right ear/left hemisphere advantage for vocal sounds using dichotic listening tasks, while a left ear/right hemisphere advantage was shown for musical sounds (for a review see Tervaniemi and Hugdahl, 2003). Whether there is an ear/hemisphere advantage for the particular sounds used in our study has yet to be shown. Another reason for false selections could be that some tones (e.g., singing bird and seagull) are more difficult to discriminate than others, therefore the combination of tones could still be optimized. Nevertheless, in Session 2 hardly any mistakes were made by the healthy participants, thus the tones are well suited for the paradigm and can possibly be better discriminated with training.

### Influences of mood and motivation

No significant influences of self-reported motivation or mood on BCI performance were found in the current study. Whiles several studies reported a positive influence of motivation on BCI Performance (Nijboer et al., 2008a, 2010; Kleih et al., 2010), other studies could not replicate this finding (Käthner et al., 2013; Kleih and Kübler, 2013).

### BCI usability

Evaluation with the SUS revealed that usability of the system is already quite good, but could still be improved. Ratings of healthy participants indicate only moderate levels of self-confidence (question 9) and ease of use of the system (question 3). However, learnability was rated as high (question 7 and 10). The overall usability rating was very high for the participant with ALS, but in terms of ease of use she also rated the system as mediocre, due to the high technical effort that is necessary to operate the system. This finding is in line with evaluations by Zickler et al. (2011, 2013), who reported that a fast, reliable and simple setup of the EEG hardware and software is necessary, if the system is to be used in daily life.

In terms of subjective workload the switch from artificial tones to animal sounds did not result in a lower score of the NASA-TLX in this study, average value 62, as compared to Käthner et al. (2013), average value 57. The participant with ALS indicated a similar workload in the first session (69) of the present study. However, it was negatively influenced by physical strain experienced during the measurements. In the second session she indicated a much lower workload (23), whereas it was still relatively high for healthy subjects in the second session (69). Workload was estimated subjectively and due to of her physical condition, we expect that the task is more difficult for the participant with ALS. Her frame of reference, on the other hand also differs from that of healthy participants, thus this task might not be as difficult for her compared to other tasks that she performs on a regular basis. A high variance between sessions for motor impaired end users was also reported in the study by Holz et al. (2013), in which participants evaluated a sensorimotor rhythm based gaming application. Workload evaluations of auditory P300 BCIs are scarce. In the single case study by Schreuder et al. (2013), the severely motor impaired end user could not achieve control over the AMUSE paradigm (Schreuder et al., 2010) and reported a high subjective workload in the first session (>90) that was lower, but still high (>60) in the subsequent sessions. To conclude, it can be stated that the task required in our study seems to be still quite difficult but not more complicated than the AMUSE paradigm. It requires users to concentrate on a particular tone in a rapidly presented sequence of tones. This is an unusual task that requires high concentration, which can, however, be learned as shown in the current study.

Compared to the classic visual row/column speller, auditory BCIs based on this paradigm impose higher workload on the user (Käthner et al., 2013). Several other authors proposed auditory spellers in which tones represent the rows and columns of the speller (Sellers and Donchin, 2006; Furdea et al., 2009; Klobassa et al., 2009; Höhne et al., 2011; Käthner et al., 2013). To keep the workload (the number of tones that need to be discriminated) at an acceptable level, a two-step procedure, similar to the one implemented in the present study, was used in several studies. The procedure of first choosing a certain row followed by a particular column to select the desired letter is used by many potential end users in a partner assisted scanning approach. The partner first presents groups of letters to the end user, who then makes a selection and in a second step chooses the letter. Therefore, this system is advantageous, once the attributions of the animal sounds to the individual rows/columns are learned. Klobassa et al. (2009) reported that all healthy participants of their study could correctly recall the assignments of tones to the corresponding rows and columns after only one or two sessions. If this is also possible for motor impaired end users, remains to be shown. In the following paragraphs, alternatives to the row/column based speller with regard to usability are discussed.

To reduce the amount of workload needed to operate an auditory multiclass BCI, Höhne and Tangermann (2014) proposed a BCI, in which voice recordings of 26 letters and four command items serve directly as target stimuli. Thus, just one step is required for making a selection and the mental effort of having to memorize sounds that represent the desired letters is eliminated. To allow for a competitive spelling speed, the alphabet was split into three groups that were each presented in a stream from a different direction over headphones (left, central (both speakers), right) and the letters of the individual groups were presented in sequential (alphabetical) order. Due to the simple instructions required to operate the system (“listen/attend to the letter you want to spell”) learnability/ease of use can be considered high. However, participants did not reach a satisfactory performance level for spelling (on average 34.7% of letters were correctly chosen across all participants). Hence, the authors of the study suggest reducing the stimulation speed, as this might improve classification accuracies.

For patients, who are unable to operate an auditory multiclass BCI or for an initial communication attempt with completely locked-in patients, paradigms that allow for a binary choice were proposed. Halder et al. (2010) tested a three-stimulus paradigm, based on the classic oddball paradigm, but including a second target. In this paradigm, users have to attend to one of two rare target tones among frequent non-target tones. With targets differing in pitch, offline analysis revealed that healthy users achieved an accuracy of 76.5% correct with two sequences (1.7 bits/min). De Vos et al. (2014) evaluated a similar paradigm with a low density, mobile EEG system and reported a maximum ITR of 1.07 bits/min with an average accuracy of 71% using one sequence. The advantage of this approach is that instructions are easy to understand (e.g., “listen/attend to the high pitched tone if you want to select yes, attend to the low pitched tone if you want to select no”) and stimuli can be presented via a single speaker, thus allowing control also for participants with deafness of one ear. Hill et al. (2004, 2005) proposed a streaming approach in which participants shift attention to one of two auditory streams presented simultaneously via headphones (left/right). In a subsequent online study, Hill and Schoelkopf (2012) showed that high online accuracies (85%) could be achieved with short stimulation intervals of a few seconds. Lopez-Gordo et al. (2012) also tested a “streaming approach” in which a dichotic listening task was employed with human voice stimuli. Although only one electrode was used (Cz), classification accuracy was high with subjects achieving 69% (1.5 bits/min). In general, these results demonstrate that the binary approaches provide reliable selection accuracies with simple to understand instructions and only low workload requirements. However, usability would decrease substantially, when trying to implement a spelling solution based on binary approaches. Users would have to complete several sub-steps to reach the desired letter which could lead to error accumulation and requires users to be aware of the current step in the spelling tree that has to be completed to reach the desired letter selection.

Binary auditory BCIs do not guarantee high spelling accuracies in situations where the windows of attention are unknown. This can be the case for participants who are in the minimally conscious state (Pokorny et al., 2013).

### Limitations

For online spelling in the second session, classification parameters of the first session were used. This is a shortcoming of the current study since offline classification accuracies using three words from Session 2 to train the classifier, yielded significantly higher accuracies in Session 2 than did the classification parameters of Session 1. On the other hand this is also an important finding, which indicates that individual parameters used for spelling can change even over a short period of time (1 day) and that contradicts (Nijboer et al., 2008b). Particularly for auditory BCIs there might be a larger variance in the individual parameters between sessions than for visual paradigms. Thus, individual classification weights were created at the beginning of both sessions for the participant with ALS. Recently Kindermans et al. (2014) proposed a probabilistic framework for BCI applications that requires no calibration run. Their zero-training approach with dynamic stopping yielded competitive results compared to a state of the art supervised method. While this was only shown in a simulated online experiment for the visual modality, the authors state that they plan to apply their approach to auditory as well as tactile BCIs. This is a promising approach that could be particularly beneficial for end users who have a limited attention span.

In this study we compared performance between the same paradigm using different stimuli across studies to estimate the effect of natural stimuli on spelling performance. While we kept most parameters, including electrode montage and classification parameters constant (same as in Käthner et al., 2013), it is likely that other factors, in particular different study participants influenced the results.

During the experiment, participants were assisted with visual aids to remember the assignment of tones to specific rows and columns. It remains to be shown in a purely auditory experiment how quickly participants can learn the assignments and use the BCI for spelling without visual aids.

## Conclusions

The study combined natural stimuli and directional cues, which have been shown to be beneficial in previous studies, with a row/column speller paradigm as a further step to optimize auditory BCIs such that they can eventually be used as assistive technology in a home environment. The study demonstrated high accuracies in two sessions that were achieved by healthy participants. Compared to a study using the same paradigm but shorter artificial stimuli (Käthner et al., 2013), an improvement of BCI performance could be observed.

Since spelling performance increased from 76 to 90% (offline) correctly selected letters in two sessions, the study also indicated a training effect that should be taken into account when testing multiclass auditory BCIs. It has yet to be shown if this effect is due to a stimulus specific learning effect (ability to discriminate the target tones) or a general adaptation to the task (familiarity with the task and the required actions for BCI control).

The training effect was also apparent during an initial test with a participant with ALS. However, the end user did not achieve accuracies sufficient for satisfactory communication, thus more training might be needed to improve spelling performance with the proposed BCI. Further improvements of the BCI could include modifications of the paradigm as well as individualized timing parameters, improvements of the stimulus material and signal processing. The effect of training in more sessions and the applicability for (completely) locked-in patients using a purely auditory paradigm without visual cues remains to be demonstrated in further studies.

### Conflict of interest statement

The authors declare that the research was conducted in the absence of any commercial or financial relationships that could be construed as a potential conflict of interest.

## Supplementary material

Supplementary Audio File 1 **Exemplary recording of the stimuli presented during one trial (selection of one letter)**.
